# Antioxidant Small Molecule Compound Chrysin Promotes the Self-Renewal of Hematopoietic Stem Cells

**DOI:** 10.3389/fphar.2020.00399

**Published:** 2020-04-02

**Authors:** Yinghui Li, Mei He, Wenshan Zhang, Ming Yang, Yahui Ding, Shiqi Xu, Jiali Gu, Yafang Li, Jingjing Yin, Yingdai Gao

**Affiliations:** ^1^ State Key Laboratory of Experimental Hematology, Institute of Hematology and Blood Diseases Hospital, Chinese Academy of Medical Sciences and Peking Union Medical College, Tianjin, China; ^2^ State Key Laboratory of Medicinal Chemical Biology, College of Pharmacy, Nankai University, Tianjin, China

**Keywords:** antioxidant, small molecule compound, chrysin, *ex vivo* expansion, hematopoietic stem cells, self-renewal

## Abstract

There is an increasing demand for the expansion of functional human hematopoietic stem cells (hHSCs) for various clinical applications. Based on our primary screening of antioxidant small molecule compounds library, a small molecule compound C2968 (chrysin) was identificated to expand cord blood CD34^+^ cells *in vitro*. Then we further verified the optimum concentration and explored its effect on hHSCs phenotype and biological function. C2968 could significantly increase the proportion and absolute number of CD34^+^CD38^−^CD49f^+^ and CD34^+^CD38^−^CD45RA^−^CD90^+^ cells under 2.5 μM. Furthermore, the total number of colony-forming units and the frequency of LT-HSCs in C2968-treated group were significantly higher than control, indicating the multipotency and long-term activity of hematopoietic stem and progenitor cells were sustained. Additionally, C2968 treatment could maintain transplantable HSCs that preserve balanced multilineage potential and promote rapid engraftment after transplantation in immunodeficient (NOG) mice. Mechanistically, the activity of chrysin might be mediated through multiple mechanisms namely delaying HSC differentiation, inhibiting ROS-activated apoptosis, and modulating of cyclin-dependent kinase inhibitors. Overall, chrysin showed good *ex vivo* expansion effect on hHSCs, which could maintain the self-renewal and multilineage differentiation potential of hHSCs. Through further research on its antioxidant mechanism, it may become a promising tool for further fundamental research and clinical umbilical cord blood transplantation of hHSCs.

## Introduction

Hematopoietic stem cells (HSCs) are a rare population of cells characterized by their ability to self-renew and differentiate into multilineages in blood system to maintain adult hematopoiesis. Hematopoietic stem cell transplantation (HSCT) is a curative therapy for a number of human diseases, including hematopoietic malignancies and bone marrow failure (Alexander et al., 2018; Pagliuca et al., 2019). However, less than half of the patients without a suitable related human leukocyte antigen (HLA)-matched donor can find an HLA-matched unrelated donor (Gragert et al., 2014). For these patients, umbilical cord blood (UCB) has become an important HSC source for allogeneic HSCT (Milano et al., 2016).The much lower immunogenicity of UCB enables transplantation despite antigen mismatch. However, the principal limitation of UCB is the low and finite number of hematopoietic stem and progenitor cells (HSPCs), which restricts their widespread use in human transplantation protocols.

To expand functional UCB HSCs available for clinical applications, researchers have been looking for suitable culture conditions to promote *ex vivo* expansion of HSC populations. Different combinations of recombinant growth factors and cytokines have been evaluated to promote HSCs proliferation but HSCs are prone to differentiate or even deplete during *in vitro* culture (Goff et al., 1998; Knapp et al., 2017). Recent studies have concentrated on applying small molecule compounds, which are gradually becoming a valuable tool for regulating the fate of stem cells. They have the virtues of simple operation, rapid and reversible effects, diverse concentrations and structures, and rapid high-throughput screening based on phenotype (Li et al., 2012; Zhang and Gao, 2016). Some of these small molecules, such as the aryl hydrocarbon antagonist StemRegenin 1 (SR1), pyrimidoindole derivative UM171, GSK-3β inhibitor CHIR99021 plus mTOR inhibitor Rapamycin (C + R), HDAC inhibitor nicotinamide (NAM), etc., have been shown to promote expansion of HSPCs in various degrees (Boitano et al., 2010; Huang et al., 2012; Peled et al., 2012; Fares et al., 2014). It is undeniable that small molecule compounds show the latent capacity for HSC expansion and stand a good chance of making a difference in this field. Our group has focused on drug screening for HSC manipulation, especially for safe expansion of HSC.

It’s essential for stem cell homeostasis to keep a proper balance between self-renewal and differentiation during both early development and the entire life cycle. Recent evidence suggests that this balance is partly regulated by reactive oxygen species (ROS), which synchronize with metabolism, mediate the redox state of cells (Miao et al., 2013; Bigarella et al., 2014; Ludin et al., 2014). It has been reported that increased ROS levels can inhibit HSC self-renewal pathways such as Wnt–β-catenin (Undi et al., 2016; Shin et al., 2004), and activate pathways that may lead to self-renewal defects such as p38 MAPK, mTOR, and more (Ito et al., 2004; Ito et al., 2006; Yoshida et al., 2011). In fact, primitive HSCs exist in a low-oxygen niche that restricts ROS production and provides long-term protection for cells (Jang and Sharkis, 2007; Dalloul, 2013). However, some researches have proved that HSCs cultured *in vitro*, together with the commonly used hydrophobic materials such as polystyrene dishes or flasks, could produce excessive level of ROS, which is a well-known cause of HSC differentiation (Bigarella et al., 2014; Yu et al., 2015; Cao et al., 2016). Therefore, strategies for maintaining hypoxia milieu to mimic HSC *in vivo* niche are important for efficient HSC expansion approaches.

In this study, we identified a small molecule compound C2968 (chrysin) with the potential for hHSC expansion based on our primary screening of 85 antioxidant small molecule compounds in LKT laboratory database (Zhang et al., 2019). Then the optimum concentration of chrysin was determined by concentration gradient experiment. We found that primitive CD34^+^CD38^−^CD49f^+^ cells and CD34^+^CD38^−^CD45RA^−^CD90^+^ cells were proportionally and quantitatively increased after culture with chrysin. We also demonstrated that chrysin expanded stem and progenitor (PRO) cell populations with sustained multipotency and long-term functional activity. Moreover, chrysin promoted rapid human cell engraftment and preserved balanced multilineage differentiation in NOG mice transplanted with expanded cells, suggesting the potential of chrysin for the *ex vivo* expansion of hHSCs with functionally validated long-term repopulating capability.

## Materials and Methods

### Experimental Animals

Female NOD/Shi-scid/IL2Rγnull (NOG) mice were purchased from Vital River Laboratory Animal Technology (Beijing, China), and were housed in a specific-pathogen-free condition, with free access to food and water. All animal protocols were approved by the Animal Care and Use Committee of State Key Laboratory of Experimental Hematology.

### Isolation of UCB CD34^+^ Cells and *In Vitro* Culture

Samples were collected from consenting donors according to ethically approved procedures at Tianjin Central Hospital of Gynecology Obstetrics (Tianjin, China). Human CD34^+^ UCB cells were isolated using LS Column and QuadroMACS Separator (Miltenyi Biotec), according to the manufacturer’s protocol after collecting CD34 MicroBead-labeled cells by Hetastarch (HES, B.Braun) and MACS CD34 MicroBeads (Miltenyi Biotec). The purity of the isolated CD34^+^ cells is about 90.58 ± 2.97% (n = 5). The absolute number of input cells was calculated based on purity and FACS data of isolated CD34^+^ cells and the initial seeding number in the culture (1 × 10^4^ CD34^+^ cells). The absolute number of output cells was obtained by viable cell count of progeny derived from 1 × 10^4^ CD34^+^ cells after culture and calculation with FACS proportion data. For phenotype and function assays, CD34^+^ cells were cultured in HSC expansion medium consisting of Iscove’s Modified Dulbecco’s Medium (IMDM, Gibco) supplemented with 10% fetal bovine serum (FBS, Gibco), 100 ng/ml human stem cell factor (SCF, PeproTech), 100 ng/ml Fms-related tyrosine kinase 3 ligand (Flt3L, PeproTech), 100 ng/ml thrombopoietin (TPO, PeproTech), and 1% Penicillin–Streptomycin (Sigma-Aldrich). Isolated CD34^+^ cells were resuspended in HSC expansion medium (5.3 × 10^4^ cells/ml) before being seeded into 96-well plates (Corning). Small molecule compounds were dissolved in dimethyl sulfoxide (DMSO, Sigma-Aldrich) and stored as stock solutions. Stock solutions were diluted to working solutions at the desired concentration by HSC expansion medium. Each well contained 190 µl cell suspension (1 × 10^4^ cells) and 10 µl small molecule compounds, which were fully blended. Cells were incubated at 37°C with 5% CO_2_ for 7 days. For transplantation experiments, human CD34^+^CD38^−^CD45RA^−^CD90^+^ cells were sorted into 96-well plates at 300 cells per well in 200 µl culture system, in which serum-free expansion medium (SFEM, StemCell Technologies) with 100 ng/ml hSCF, 100 ng/ml TPO, and 100 ng/ml Flt3L was supplemented with vehicle (0.01% DMSO) or SR1 (Alichem, 41864) [1 µM] or Chrysin (C2968, purity ≥98%, LKT Labs) [2.5 µM]. Cells were incubated at 37°C with 5% CO_2_ for 4 days.

### Flow Cytometric Analysis

CD34^+^ UCB cells were seeded at 1 × 10^4^ cells per well in the presence of chemical compounds. Relative percentages and absolute numbers of different HSC subsets were determined after 7 days of culture using flow cytometry. Cell phenotypes in expanded cells were stained at 4°C for 30–60 min in phosphate-buffered saline (PBS) supplemented with a combination of the following antibodies and fluorophores: APC-labeled anti-human CD34 (BD; 555824), PE-Cy7-labeled anti-human CD38 (BD; 560677), APC-H7-labeled anti-human CD45RA (BD; 560674), PerCP-Cy5.5-labeled anti-human CD90 (BD; 561557), and PE-labeled anti-human CD49f (BD; 555736). Following a wash step, stained cells were analyzed using LSRII flow cytometer (BD) and FlowJo 10 software.

### Colony-Forming Cell Assays

The concentration of cultured CD34^+^ UCB cells was adjusted to 50 µl/1000 initial cells in IMDM. Frequencies of colony-forming cells (CFC) were estimated by plating 10 µl cell suspension (equivalent to 200 initial cells) in 1 ml MethoCult H4434 Classic (StemCell Technologies) in six-well plates (Corning). After 14 days in culture, plates were visually scored for CFU-GM, CFU-E, BFU-E, and CFU-GEMM.

### Cobblestone Area-Forming Cell Assays

Cultured CD34^+^ UCB cells were resuspended with MyeloCult H5100 long-term culture medium (Stem Cell Technologies) supplemented with 10^−6^ mol/L hydrocortisone (Sigma-Aldrich) and seeded on irradiated (8000 cGy) M2-10B4 bone marrow stromal cells (ATCC) in flat-bottomed collagen-coated 96-well plates at five different concentrations (63, 125, 250, 500, 1000) with 12 replicates per dilution. Each well contained 50 µl collagen (Stem Cell Technologies) and 1.2 × 10^4^ M2-10B4 cells. After 5 weeks of culture, all wells were scored microscopically. Wells were scored as being positive for the presence of at least one cobblestone area (CA, tightly knit group of phase-dark, angular cells in the stroma). The CA-forming cell (CAFC) frequencies were calculated with the Poisson formula from the percentage of negative wells using the L-Calc software (Stem Cell Technologies).

### Transplantation and Monitoring of Human HSCs in NOG Mice

At 6 to 7 weeks of age, mice were irradiated at a dose of 250 cGy 4 h prior to transplantation. Experiments were conducted in sodium pentobarbital-anesthetized mice. For uncultured group, freshly sorted UCB DAPI^−^CD34^+^CD38^−^CD45RA^−^CD90^+^ cells were counted and resuspended in 300 cells/25 µl PBS per mouse, and injected into mouse tibiae. For small molecules or DMSO-treated groups, 300 sorted DAPI^−^CD34^+^CD38^−^CD45RA^−^CD90^+^ cells were cultured for 4 days in serum-free expansion system with the presence of C2968 or SR1 or DMSO as previously described. The expanded bulk-cell cultures were washed by PBS and injected into mouse tibiae in 25 µl PBS. Human cell chimerism was analyzed at 4, 8, and 12 weeks post-transplantation in the peripheral blood and 16 weeks post-transplantation in the injected side and opposite side of bone marrow, using FITC-labeled anti-human CD45 (BD; 555482). Multilineage reconstitution was analyzed in bone marrow using APC-Cy7-labeled anti-human CD33 (BioLegend; 366613), PerCP-Cy5.5-labeled anti-human CD19 (BD; 561295), PE-Cy7-labeled anti-human CD3 (BD; 557851), PerCP-Cy5.5-labeled anti-human CD56 (BD; 560842), APC-labeled anti-human CD235a (BD; 561775), and PE-labeled anti-human CD41a (BD; 557297).

### Single Cell PCR

The following four populations were collected by Influx flow cytometry (BD) after culture: HSCs (CD34^+^CD38^−^CD45RA^−^CD90^+^CD49f^+^), HSPCs (CD34^+^CD38^−^), multipotential progenitor cells (MPPs, CD34^+^CD38^−^CD45RA^−^CD90^−^) and progenitor cells (PROs, CD34^+^CD38^+^). Total RNA was extracted from each population using a CellsDirect One-Step qRT-PCR Kit (Invitrogen) according to the manufacturer’s instructions. Briefly, 50 cells from each population were sorted directly into a mixture of CellsDirect 2× Reaction Mix, 0.2× TaqMan Assay Mix (Applied Biosystems), and SuperScript III RT/Platinum Taq Mix (Invitrogen). Reverse transcription (RT) and specific target amplification (STA) were serially performed with the following parameters: 15 min at 50°C, 2 min at 95°C, and 18 cycles of 95°C for 15 s and 60°C for 4 min. Preamplified cDNA was then diluted with TE buffer (1:5) and subjected to real-time PCR. Briefly, a BioMark 96·96 Dynamic Array (Fluidigm) was used and the PCR parameters were: 10 min at 95°C, followed by 40 cycles of 15 s at 95°C and 60 s at 60°C. Data were analyzed using BioMark Real-Time PCR Analysis Software (Fluidigm, USA).

### Statistical Analysis

All data are presented as the mean ± standard deviation (SD) and all statistical analyses were done using the software Graphpad Prism version 7.0 (GraphPad Software, Inc., La Jolla, CA, USA). Statistical differences were evaluated using two tailed Student’s t-test, with significance at p values ≤ 0.05.

## Results

### Identification of the Optimum Working Concentration of Chrysin

**Figure 1:**
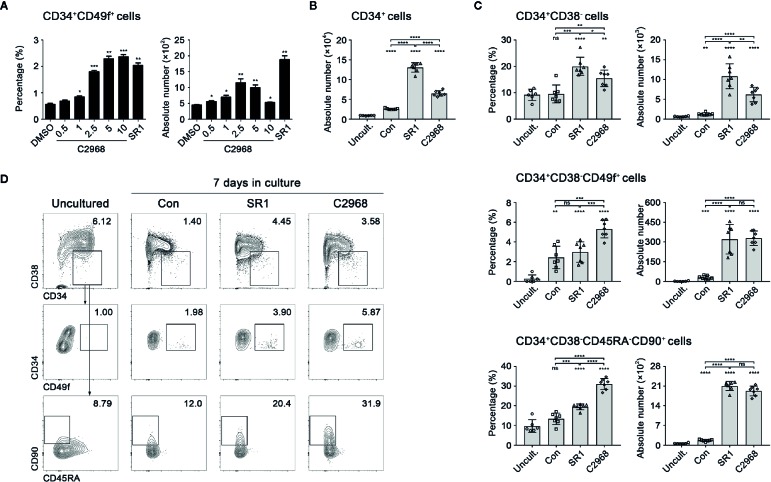
Chrysin promoted *in vitro* expansion of primitive human hematopoietic stem and progenitor cells. **(A)** The percentages and absolute numbers of CD34^+^CD49f^+^ cells after 7 days in cultures supplemented with concentration gradient (0.5, 1, 2.5, 5, and 10 µM) of C2968 (n = 5). The concentration of SR1 was 1 µM. **(B)** Quantification of total input and output CD34^+^ cells in culture (n = 7; n = 6 for uncultured cells). UCB CD34^+^ cells (1 × 10^4^) were seeded in the beginning of the culture. **(C)** The percentages and absolute numbers of CD34^+^CD38^−^, CD34^+^CD38^−^CD49f^+^, and CD34^+^CD38^−^CD45RA^−^CD90^+^ cells derived from uncultured CD34^+^ cells or after 7 days in cultures supplemented with Con [dimethyl sulfoxide (DMSO) 0.01%], SR1 (1 µM), and C2968 (2.5 µM) (n = 7; n = 6 for uncultured cells). All data represent the means ± SD. Compared with uncultured group unless specified, *p < 0.05, **p < 0.01, ***p < 0.001, ****p < 0.0001 and ns, no significance by two-tailed unpaired t-test. ○, □, Δ and ◊ represent a single data point in Uncultured (Uncult.), Con, SR1 and C2968 groups respectively. **(D)** Representative FACS profiles of CD34^+^CD38^−^, CD34^+^CD38^−^CD49f^+^, and CD34^+^CD38^−^CD45RA^−^CD90^+^ populations derived from total live cells in uncultured and cultured CD34^+^ cells.

Based on our preliminary screening of 85 antioxidant small molecule compounds in database of LKT laboratory (Zhang et al., 2019), 0.5 µM C2968 was initially screened out as an active candidate compound. To investigate the optimum concentration of C2968, primary human CD34^+^ cells isolated from UCB were seeded into 96-well plates (1 × 10^4^ cells per well) in expansion medium supplemented with SCF, TPO, and Flt3L. C2968 was added at a concentration gradient. The cells were cultivated for 7 days, then the mixture of expanded cells were analyzed by flow cytometry. We identified C2968 as a candidate promoter of HSPCs self-renewal, with effective concentrations of 0.5 to 10 µM when tested for its ability to stimulate the expansion of a more primitive HSPC populations, CD34^+^CD49f^+^ cells. The proportion of CD34^+^CD49f^+^ cells was positively correlated with the concentration of C2968. As for the absolute number, the best effect was obtained at 2.5 µM, which induced a 2.6-fold increase in the absolute number of CD34^+^CD49f^+^ cells when compared with control group (DMSO) (**Figure 1A**). Therefore, the optimum working concentration of chrysin was identified as 2.5 µM.

### Chrysin Treatment of UCB CD34^+^ Cells *In Vitro* Led to Preferential Expansion of Primitive HSCs

It is known that CD34, CD38, CD90, CD45RA, and CD49f are common cell surface markers for identification of human HSCs and PRO cells *in vitro* and *in vivo*, and the measure of different combination of these markers is a close estimate of HSPCs (Notta et al., 2011). To further evaluate the ability of C2968 to expand HSPCs *in vitro*, we compared the impacts of C2968, SR1, and Control on outputs of UCB CD34^+^ cells in expansion medium for 7 days. The absolute numbers and percentages of HSPCs and HSCs were determined according to their immunophenotypes: CD34^+^CD38^−^ (HSPCs), CD34^+^CD38^−^CD49f^+^ (HSCs) and CD34^+^CD38^−^CD45RA^−^CD90^+^ (HSCs). The total outputs of CD34^+^ cells were significantly increased after culture, in which Con (0.01% DMSO), SR1, and C2968 expanded 2.8-, 14.4-, and 7.2-fold greater than input respectively (**Figure 1B**). As for CD34^+^CD38^−^ cells, both SR1 and C2968 promoted a higher percentage and absolute number than Con or the uncultured same lot HSPCs, with better performance in the presence of SR1. As for the more primitive subsets, CD34^+^CD38^−^CD49f^+^ cells and CD34^+^CD38^−^CD45RA^−^CD90^+^ cells, it was proportionally more abundant when C2968 was present. Both SR1 and C2968 achieved significantly greater HSC quantities, with no significant difference between two compounds (**Figures 1C, D**). These findings indicated that chrysin supported preferential expansion of primitive human HSCs *in vitro*.

### Chrysin Treatment Sustained Multipotency and Long-Term Activity of HSPCs *In Vitro* Culture

**Figure 2:**
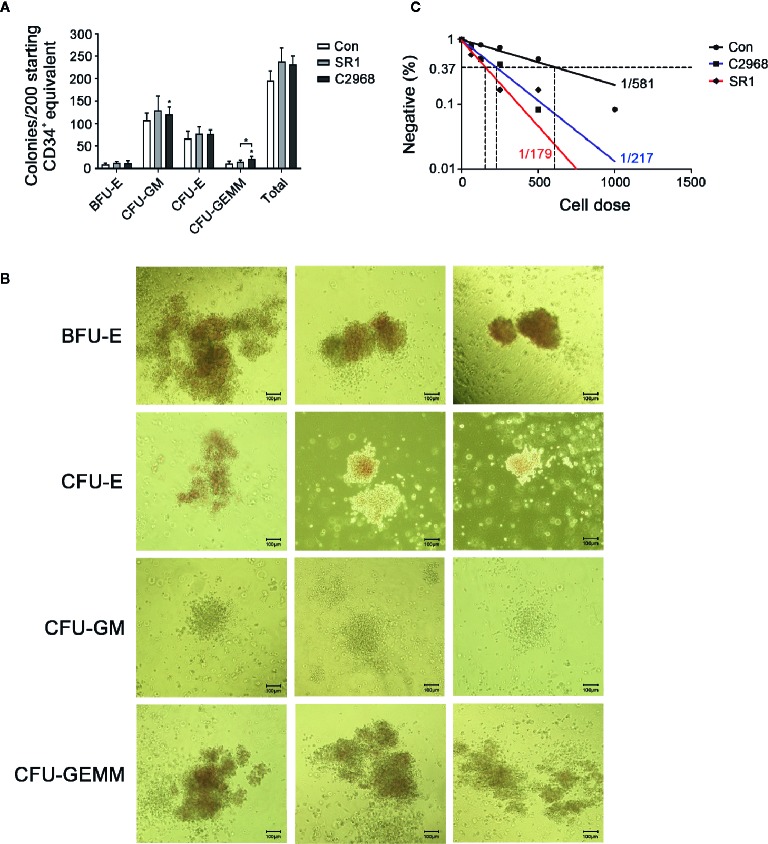
Chrysin-treated CD34^+^ cells sustained *in vitro* multiple-differentiation potential and long-term hematopoietic capacity in culture. **(A)** Colonies derived from multipotent progenitor cells (BFU-E, burst-forming unit-erythroid; CFU-GM, colony-forming unit-granulocyte/macrophage; CFU-E, colony-forming unit-erythrocyte; CFU-GEMMs, colony-forming unit-granulocyte/erythrocyte/macrophage/megakaryocyte) after 14 days in H4434 methylcellulose culture (n = 4). **(B)** Representative morphological images of CFU colonies described in **(A)**. **(C)** Frequency of long-term repopulating cells within Con (DMSO 0.01%), SR1 (1 µM), or C2968 (2.5 µM) cultures measured by LDA. Positive well was defined as having more than one cobblestone-like primitive hematopoietic area. All data represent the means ± SD. Compared with control unless specified, *p < 0.05 by two-tailed unpaired t-test.

One major challenge of HSC expansion *ex vivo* is that the culture of HSCs results in a loss of multipotency. So we next asked whether cells treated with C2968 have the ability to emerge multiple lineage cells after culture. We performed CFC assay after 7 days of culture and approximately 1/50th of the resulting populations (about 200 starting CD34^+^ equivalent) were transferred to semisolid differentiation medium for another 14 days to allow colony formation. C2968-treated cells showed a prominent colony-forming potential of granulocyte and macrophage (GM) when compared to control. A comparable increase was also observed in granulocyte–erythrocyte-macrophage–megakaryocyte (GEMM) colonies, which reflected the function of MPPs, when treated with C2968 instead of SR1. As for erythroid colonies, the number of BFU-E and CFU-E were almost the same between C2968 and SR1 groups (**Figure 2A**). Representative morphological images of different types of colonies are shown in **Figure 2B**. These results indicated that chrysin retained the capacity of HSPCs to differentiate into multilineages in cell cultures.

We also assessed the long-term functional capacity of C2968-treated cells in CAFC assay. Cultured UCB CD34^+^ cells were planted in flat-bottomed collagen-coated 96-well plates pre-added with M2-10B4 stromal cells at limiting doses (63, 125, 250, 500, 1000 cells). The culture lasted for 5 weeks and half of the H5100 medium was changed every week during this period. LDA revealed that 0.46% (1 in 217) of C2968-treated CD34^+^ cells had long-term activity, which was closer to 0.56% (1 in 179) of SR1 as compared with 0.17% (1 in 581) of Control (**Figure 2C**). These results indicated that chrysin maintained the identity and long-term viability of cultured HSPCs without blocking differentiation. Together, these observations showed that our chrysin mediated *in vitro* CD34^+^ cell culture technique enabled HSPCs expansion with sustained multipotentiality.

### Chrysin Retained Human UCB HSC Engraftment Capacity and Multilineages Differentiation Potential in NOG Mice

**Figure 3:**
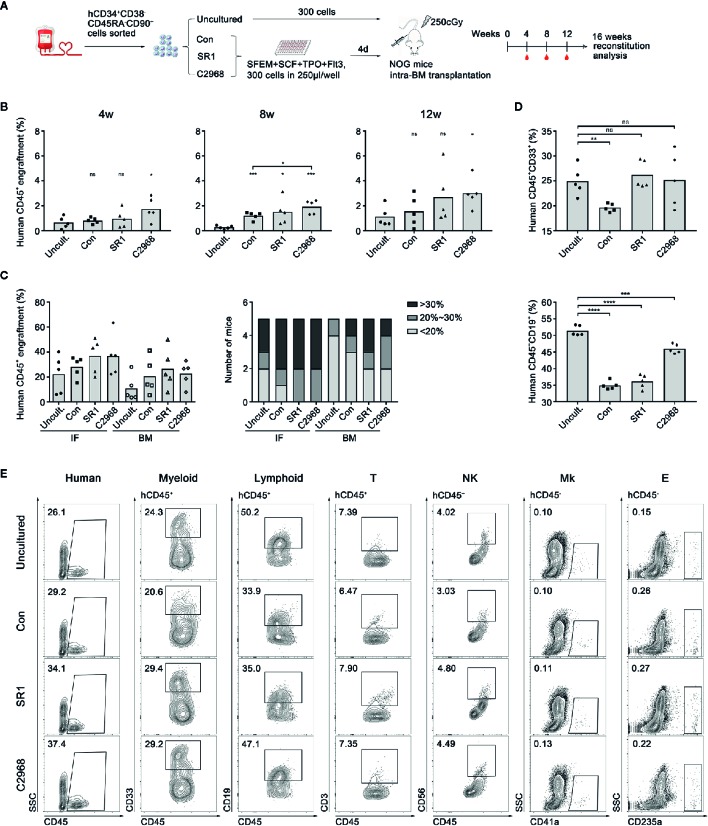
Analysis of the effect of chrysin treatment on human hematopoietic cell engraftment and reconstitution in NOG mice. **(A)** Transplantation strategy with uncultured or *ex vivo* expanded human CD34^+^CD38^−^CD45RA^−^CD90^+^ cells. Uncultured cells were transplanted into NOG mice on day 0 and cultured cells derived from 300 input cells were transplanted in to NOG mice on day 4. **(B)** Levels of human cell engraftment in peripheral blood (PB) of NOG mice at 4, 8, or 12 weeks post-transplantation. (n = 5 mice per group) **(C)** Levels of the engrafting human cells (hCD45^+^) in the injection side bone marrow (IF) and the opposite side bone marrow (BM) at 16 weeks post-transplantation. Number of mice at different engraftment levels (< 20%; 20%~30%; > 30%) in each group were shown. **(D)** Levels of the reconstituting myeloid (hCD45^+^ CD33^+^) and lymphoid (hCD45^+^ CD19^+^) in the injection side and the opposite side bone marrow at 16 weeks post-transplantation. **(E)** Representative flow cytometric profiles of the engrafting human CD45^+^ cell and myeloid, lymphoid, T lymphocyte (hCD45^+^ CD3^+^), natural killer cell (NK, hCD45^+^ CD56^+^), megakaryocyte (Mk, hCD45^−^ CD41a^+^), and erythroid (E, hCD45^−^ CD235a^+^) reconstitution in BM 16 weeks post-transplantation. All data represent the means ± SD. Compared with uncultured group unless specified, *p < 0.05, ***p < 0.001, ****p < 0.0001 and ns, no significance by two-tailed unpaired t-test.●, ■, ▲ and ♦ represent a single data point in Uncultured (Uncult.), Con, SR1 and C2968 groups respectively in **(B, D)**. ●, ■, ▲ and ♦ represent a single data point of IF in Uncult., Con, SR1 and C2968 groups respectively while ○, □, Δ and ◊ represent those of BM in **(C)**.

Xenotransplantation into immunodeficient mice has been the gold standard assay to detect the function of HSCs *in vivo*. To further expand on our observation that chrysin promoted expansion of human UCB CD34^+^ cells and examine its effect on hematopoietic reconstitution, we conducted transplantation assay in severe combined immunodeficient NOD/Shi-scid/IL2Rγ^null^ (NOG) mice (**Figure 3A**). For uncultured group, 300 human UCB CD34^+^CD38^−^CD45RA^−^CD90^+^ cells were freshly sorted and transplanted directly into NOG mice. For compound-treated groups, the same amount of cells was sorted and cultured in clinically suitable conditions using SFEM with C2968, SR1, or vehicle control (DMSO) for 4 days. Then the cultured progeny derived from 300 HSCs were transplanted to recipients by intramedullary injection (n = 5). C2968-treated cells showed slightly higher level of human CD45^+^ cells engraftment in peripheral blood of recipient mice than that of uncultured group at the time point of 4 or 12 weeks post-transplantation. At week 8, the engraftment achieved significantly higher than uncultured or control group following treatment with C2968. However, SR1 group only showed a higher engraftment than uncultured group at week 8. These results indicated that chrysin might promote rapid engraftment after transplantation *in vivo* (**Figure 3B**). To explore the long-term reconstitution and migration of transplanted cells, the implantation rates in bone marrow of the injection side (IF) and non-injection side (BM) were evaluated at 16 weeks post-transplantation. There would be some slight distinction between compound-treated groups and others if we focus on positive engraftment with higher donor chimerism rate. As for the injection side (IF), human CD45^+^ engraftment of all recipients in C2968 group achieved over 20%, as was the case in SR1group, while 4/5 mice in control and 3/5 mice in uncultured group achieved over 20%. As for the non-injection side (BM), the engraftment trends were consistent with IF, with 3/5 in C2968 and SR1 group, 2/5 in control, and 1/5 in uncultured group achieving over 20% (**Figure 3C**). We also observed that C2968-treated cells could differentiate into multiple lineages including myeloid (CD45^+^CD33^+^), T lymphoid (CD45^+^CD3^+^), natural killer cell (CD45^+^CD56^+^), megakaryocyte (CD45^−^CD41a^+^), and erythoid (CD45^−^CD235a^+^) after transplantation without significant difference compared to uncultured cells (**Figures 3D, E**). Quantification of B lymphoid differentiation in engrafted mice confirmed a more comparable reconstitution characteristics of C2968-treated and uncultured HSPCs (**Figure 3D**). Accordingly, this finding suggested that chrysin might promote rapid engraftment after transplantation, which is good for faster recovery of hematopoietic reconstitution, and sustain transplantable HSCs that preserve homing and balanced multilineage potential during culture.

### Chrysin May Promote Self-Renewal of HSCs by Maintenance of Quiescence and Inhibition of Differentiation Through Multiple Pathways

**Figure 4:**
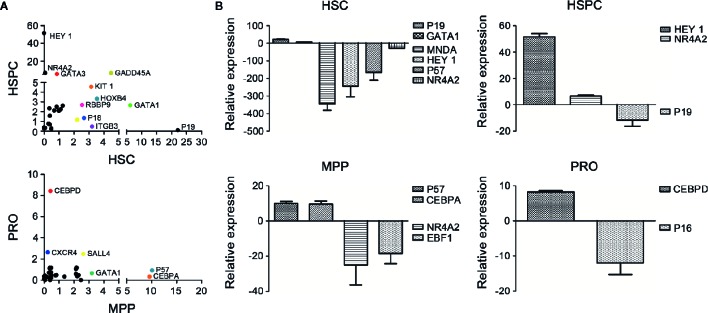
Differential expression analysis of the genes related to hematopoiesis in cultured hematopoietic cells with or without chrysin treatment. **(A)** Scatter plot of mRNA profile showing upregulated genes in cultured HSCs (26 genes), HSPCs (26 genes), MPPs (28 genes), and PROs (26 genes) with C2968 treatment. The genes exhibiting significant changes in expression compared with the control group are labeled (p < 0.001). **(B)** Fold changes of relative mRNA expression of the most significant upregulated or downregulated genes in cultured HSCs, HSPCs, MPPs, or PROs with C2968 treatment. All data were normalized to the expression levels in control (DMSO 0.01%) group. Graphs show means ± SD (n = 100 cells). HSCs, hematopoietic stem cells; HSPCs, hematopoietic stem and progenitor cells; MPPs, multipotential progenitor cells; PROs, progenitor cells.

Under steady-state conditions, HSCs stay in dormancy featured with slow cell cycle, such as quiescent phase (Pietras et al., 2011). In reaction to external stimulus, HSCs quit the G0 phase and make a variety of fate choices, such as self-renewal, multilineage differentiation, apoptosis or aging, which are controlled by stem cell intrinsic regulators as well as external modulators mainly provided by microenvironment (Wilson et al., 2009; Nakamura-Ishizu et al., 2014). To assess potential changes in proliferation and differentiation related gene expression in HSCs after C2968 treatment, we performed single cell PCR comparing different HSPC subsets sorted from UCB CD34^+^ cells cultured for 7 days with C2968 and those with Control (0.01%DMSO). Each sample was assayed by PCR to detect expression levels of 95 genes. Approximately one third of these genes were found to be up-regulated following C2968 treatment in all four cell populations (**Figure 4A**). The mRNA profiling revealed that the cell cycle inhibitor p19, which participated in the regulation of HSC quiescence by inhibiting cell cycle transition of G0/G1 (Hilpert et al., 2014), exhibited 22-fold increase in gene expression in C2968-treated HSCs (CD34^+^CD38^−^CD45RA^−^CD90CD49f^+^) compared to the control group (**Figure 4B**). Moreover, HoxB4 has been shown to be a strong positive regulator of HSC self-renewal (Antonchuk et al., 2002; Beslu et al., 2004), and we found that HoxB4 expressed at a significantly higher level in C2968-treated HSCs compared with control (**Figure 4A**). Nuclear Receptor Subfamily 4 Group A Member 2 (NR4A2) has been proved to regulate differentiation, proliferation and migration of various stem cells (Sirin et al., 2010; Maijenburg et al., 2012), and NR4A2 knockdown inhibited ROS-activated autophagy-dependent apoptosis in stem cells (Shi et al., 2017). C2968 treatment resulted in significant down-regulation of pro-apoptotic NR4A2 by 13-fold in HSCs, which might result in HSC self-renewal and expansion. The level of differentiation-related transcription factors, such as MNDA and HEY1, were significantly reduced by 337- and 217-fold respectively in HSCs (**Figure 4B**). C2968-treated HSPCs (CD34^+^CD38^−^), MPPs (CD34^+^CD38^−^CD45RA^−^CD90^−^), and PROs (CD34^+^CD38^+^) had significantly increased expression of lineage-priming factors, including HEY1, GATA3, CEBPA, and CEBPD by 51-, 5-, 9-, and 8-fold respectively (**Figures 4A, B**). Combined with our *in vitro* and *in vivo* experiments, these data may likely provide some clues about how chrysin played its role in HSC expansion. While none of the effect alone may be enough to promote the self-renewal of HSC, in combination these pathways could provide the compositions requisite for self-renewal: proliferation while blocking differentiation and preventing apoptosis.

## Discussion

The *ex vivo* expansion of UCB-derived HSCs is an increasingly popular technique to improve the therapeutic effect of HSCT. Among numerous strategies proposed, cytokines are the most fundamental amplification tool such as classic combination of SCF, TPO, Flt3-L, and interleukins (Pineault et al., 2011), others like Delta-1, Jaggad-1, SALL4, and TAT-BMI-1 (Codispoti et al., 2017). In addition, small molecules, which are basically found through mass screening, are effective agonists for *in vitro* amplification of UCB HSCs. For example, the latest discoveries are antagonist of PPAR-gamma GW9662 (Guo et al., 2018), inhibitor JNK-IN-8 (Xiao et al., 2019), and so on. Moreover, it is found that some co-culture systems or materials could provide a suitable microenvironment for HSC expansion, such as co-culture with polyvinyl alcohol (PVA) (Wilkinson et al., 2019), zwitterionic hydrogel (Bai et al., 2019), or mesenchymal stem/stromal cells (Costa et al., 2018). Since HSCs are deemed to grow in hypoxic environment (Takubo et al., 2010), low oxygen also plays an important role in the *in vitro* expansion of HSC. Under the condition of hypoxia, the expression of CXCR4 statistically increased, which means the ability of homing increased (Mohammadali et al., 2018). The use of p38-MAPK inhibitor (Bari et al., 2018) or antioxidants can maintain low ROS levels, protect HSCs from oxidative stress, and inhibit cell aging and differentiation (Ludin et al., 2014; Cao et al., 2016; Testa et al., 2016). This suggests that antioxidant small molecules may be of potential development value for expansion of HSCs, which can scavenge excessive ROS and repair oxidative damage, thus protecting the biological function of stem cells such as promoting engraftment of HSCs (Naka et al., 2008; Hao et al., 2014; Hu et al., 2014).

Based on our large-scale preliminary screening of 85 antioxidant small molecule compounds in the earlier stage (Zhang et al., 2019), we selected chrysin (C2968), which showed a better effect of expanding HSCs *in vitro*, for further study. Chrysin (5,7-dihydroxyflavone), a kind of natural flavone, exists in a number of plant extracts including propolis and honey. It is one of the most widely used herbal active ingredient in Asian countries. Nowadays, chrysin has become a valuable candidate exhibiting with multiple biological activities such as antioxidant, anti-inflammatory, anti-allergic, anti-diabetic, antibacterial, and so on (Kasala et al., 2015; Mani and Natesan, 2018). Researchers have investigated the structure–activity relationship of chrysin to clarify the mechanism of action of chrysin, suggesting its effect on multiple signaling pathways such as Wnt, NF-κB, and PIK3 (Kasala et al., 2015; Mani and Natesan, 2018), which are also closely related to stem cells regulation. In recent years, there are a growing number of researches on chrysin and its derivatives. Some progress has been made in the effect of chrysin on the promotion of mesenchymal stem cell proliferation and maintenance of human adipose-derived stem cells stemness (Deldar et al., 2017; Menon et al., 2018). However, in the field of hematopoiesis, the effect of chrysin on HSCs has not been reported.

In this study, we demonstrated that chrysin was effective in the *ex vivo* expansion of human UCB HSCs especially more primitive CD34^+^CD38^−^CD49f^+^ and CD34^+^CD38^−^CD45RA^−^CD90^+^ cells. A comparable increase in GM and GEMM colonies in CFC assay reflected that chrysin promoted the self-renewal ability of HSPCs meanwhile retained the capacity to differentiate. It’s known that a high dose of functional hematopoietic PRO cells, which often measured as CFU-GM, in the UCB graft predict an increased likelihood of successful engraftment, and faster times to neutrophil and platelet recovery (Avery et al., 2011). An increased frequency of LT-HSCs in CAFC assay suggested that chrysin expanded HSCs with long-term activity in culture. The *in vivo* data further supported chrysin played a part in promoting rapid engraftment after transplantation and retaining human cell engraftment ability and multilineage hematopoiesis capacity without bias. Single cell PCR analysis suggested that the increase in the number of functional HSCs was assumed to be the result of chrysin inhibiting differentiation and preventing apoptosis. It is known that CDKIs control the cell cycle directly through inhibiting cell cycle entry, which involve in the regulation of HSC quiescence and play an important role in maintenance of HSCs. We found that the expression of the Ink4 family member of cyclin-dependent kinase inhibitors (CKIs) p19 (Hilpert et al., 2014) increased markedly in C2968-treated HSCs, meanwhile the Kip family member of CKIs p57 (Matsumoto et al., 2011) showed different trends in C2968-treated HSCs and MPPs. It suggested that chrysin may help to preserve a quiescent, multipotential HSC pool that intermittently yielded multipotent PROs with robust proliferative potential to sustain hematopoiesis and short-term rescue. In addition, we found that a strong positive regulator of HSC self-renewal HoxB4 (Antonchuk et al., 2002; Beslu et al., 2004) was highly expressed in C2968-treated HSCs. At the differentiation level, chrysin treatment resulted in reduced expression of differentiation-related transcription factors such as human hematopoietic cell specific nuclear protein MNDA, which is associated with myeloid lineage differentiation and HSC quiescence (Xie et al., 1998; Lu et al., 2018), and Notch signaling downstream effector HEY1 (Sharff et al., 2009) in HSCs. On the contrary, chrysin-treated HSPCs, MPPs, and PROs had significantly increased expression of lineage-priming factors, including HEY1, GATA3, CEBPA, and CEBPD. Mechanistically, nuclear receptor transcription factor NR4A2 knockdown inhibited ROS-activated autophagy-dependent apoptosis in stem cells (Shi et al., 2017) and NR4A2 was also a limiting factor of the proliferation of HSCs (Sirin et al., 2010). Chrysin treatment resulted in reduced expression of pro-apoptotic NR4A2 in HSCs, which may result in HSC self-renewal and expansion. The molecular mechanism underlying the pleiotropic activities of chrysin was diverse, which may involve combinations of multi-levels of hematopoietic cell signaling pathways.

## Conclusion

*Ex vivo* expansion of UCB-derived HSCs without causing differentiation into mature cells is considered to be an efficient procedure that is able to alter clinical treatments by making available UCB units and improving transplantation-related outcomes. Accordingly, cytokine combinations, addition of small molecules, O_2_ level, co-culture systems, as well as gene manipulation of HSCs can have effects on their expansion and growth. However, past many efforts mainly target single or limited pathways and often lead to lineage bias or expansion of PRO cells or limited LT-HSCs. We believe that the real way out in the future is to target multiple pathways required for maintaining HSCs through the combinations of different molecules or methods, thus resulting in expansion of functional LT-HSCs. As a promising antioxidant small molecule, chrysin-treated hHSCs could maintain long-term hematopoiesis and differentiate into multilineages of hematopoietic cells in immunodeficient mice, which provided evidence that chrysin induced unique *ex vivo* expansion of hHSCs without carcinogenicity or severe toxicological effects. Taken together, we herein provide a potential approach for promoting self-renewal of UCB-derived HSCs *ex vivo*, which provides research basis and available choice for expansion strategies combination.

## Data Availability Statement

All datasets generated for this study are included in the article/supplementary material.

## Ethics Statement

The studies involving human participants were reviewed and approved by Tianjin Central Hospital of Gynecology Obstetrics. The patients/participants provided their written informed consent to participate in this study. The animal study was reviewed and approved by Animal Care and Use Committee of State Key Laboratory of Experimental Hematology. Written informed consent was obtained from the individual(s) for the publication of any potentially identifiable images or data included in this article.

## Author Contributions

MH performed research, interpreted the data, and wrote the article. WZ prepared UCB CD34^+^ cells. JG, SX, YFL, JY, and YD performed research. MY carried out work on mice. YHL was responsible for critical reading of the manuscript and important intellectual content. YG was responsible for the study concept, design, and execution of the research, interpretation of data, and revision of the draft paper.

## Funding

This work was supported by grants from the Ministry of Science and Technology of China (Nos. 2016YFA0100600 and 2017YFA0104900), the National Natural Science Foundation of China (NSFC 81870083, 81430004, 81421002, and 81970105), CAMS Innovation Fund for Medical Sciences (Nos. 2016-I2M-3-008 and 2016-I2M-1-017), Tianjin Science and Technology Planning Project (No. 18ZXXYSY00010), and a SKLEH-Pilot Research Grant.

## Conflict of Interest

The authors declare that the research was conducted in the absence of any commercial or financial relationships that could be construed as a potential conflict of interest.
